# Supplementary material for: Identification of photoperiod-induced specific miRNAs in the adrenal glands of Sunite sheep (Ovis aries)
Source: Front Vet Sci. 2022 Jul 22;9:888207. doi: 10.3389/fvets.2022.888207 (PMC9354845; doi:10.3389/fvets.2022.888207)
Supplement: Supplementary file 1 [file Data_Sheet_1.ZIP › Supplementary file/miRDeep2 file.zip!/miRDeep2 file/Novel_Structure/LP42A1/Novel_563.pdf]

Provisional ID	:	NC_019475.2_27254
Score total	:	1
Score for star read(s)	:	-1.3
Score for read counts	:	0
Score for mfe	:	0.7
Score for randfold	:	1.6
Score for cons. seed	:	
Total read count	:	1163
Mature read count	:	1163
Loop read count	:	0
Star read count	:	0

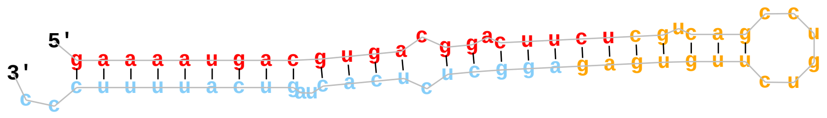

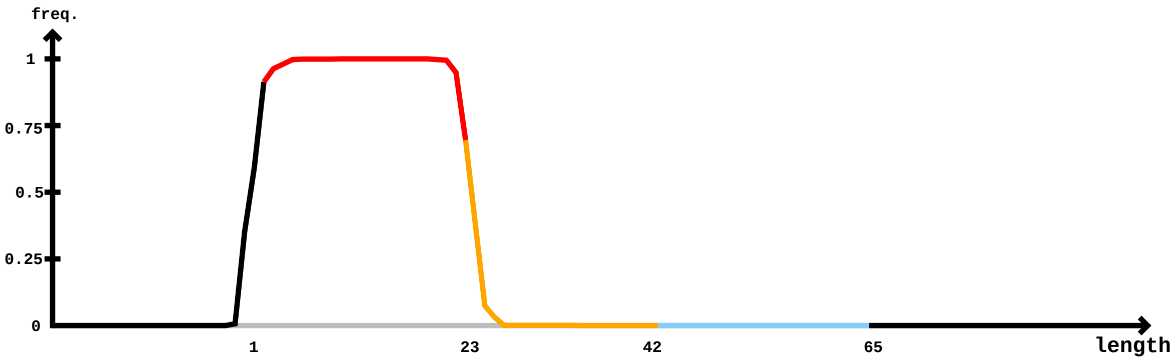

Mature Star

```
5'- gauacgugaaaaggcgucuugaaaaugacgugacggacuucucgucagccugucuugugagaggcucucacuagucauuuucccaccaauguccugcuuucagagguuucau   -3'   exp
    ((.((.(((((((((((...(((((((((((((.((.(((((((.(((......))))))))))))).))))..))))))))......))).)))..)))))...)).))..   reads   mm   sample
    .................cuugaaaaugacgugacggac..........................................................................   1       0    seq
    .................cuugaaaaugacgugacggacu.........................................................................   2       0    seq
    .................cuugaaaaugacgugacggacuuc.......................................................................   2       0    seq
    .................cuugaaaaugacgugacggacuucu......................................................................   3       0    seq
    ..................uugaaaaugacgugacggac..........................................................................   2       0    seq
    ..................uugaaaaugacgugacggacuu........................................................................   54      0    seq
    ..................uugaaaaugacgugacggacuucu......................................................................   349     0    seq
    ...................ugaaaaugacgugacggacuuc.......................................................................   270     0    seq
    ...................ugaaaaugacgugacggacuucuc.....................................................................   9       0    seq
    ...................ugaaaaugacgugacggacuucucg....................................................................   1       0    seq
    ....................gaaaaugacgugacggacu.........................................................................   1       0    seq
    ....................gaaaaugacgugacggacuuc.......................................................................   26      0    seq
    ....................gaaaaugacgugacggacuucu......................................................................   351     0    seq
    .....................aaaaugacgugacggacuucuc.....................................................................   9       0    seq
    .....................aaaaugacgugacggacuucucg....................................................................   34      0    seq
    .....................aaaaugacgugacggacuucucgu...................................................................   15      0    seq
    ......................aaaugacgugacggacuucu......................................................................   2       0    seq
    ......................aaaugacgugacggacuucuc.....................................................................   3       0    seq
    ......................aaaugacgugacggacuucucg....................................................................   15      0    seq
    .......................aaugacgugacggacuucu......................................................................   1       0    seq
    .......................aaugacgugacggacuucucgu...................................................................   19      0    seq
    ........................augacgugacggacuucucgu...................................................................   2       0    seq
    ............................cgugacggacuucucgucagccugu...........................................................   1       0    seq
```
